# Efficacy of External Siddha Pharmacotherapy for Giant Acrochordon: A Case Report

**DOI:** 10.7759/cureus.84012

**Published:** 2025-05-13

**Authors:** Saravanasingh Karan Chand Mohan Singh, Sugumaran Pichamuthu, V Gowri, Murugesan Sannasi, Chenthamarai Selvi G

**Affiliations:** 1 Department of Maruthuvam, National Institute of Siddha, Chennai, IND; 2 Department of Special Medicine, Government Siddha Medical College, Chennai, IND; 3 Department of Gunapadam, Maria Siddha Medical College and Hospital, Thiruvattar, IND; 4 Department of Nanju Maruthuvam (Siddha Toxicology), National Institute of Siddha, Chennai, IND; 5 Department of Sattam Sarntha Maruthuvam and Nanju Maruthuvam, Government Siddha Medical College Palayamkottai, Tirunelveli, IND

**Keywords:** acrochordon, external, pachaieruvai, siddha medicine, skin tag

## Abstract

Giant acrochordons, commonly referred to as skin tags, are benign, pedunculated lesions often linked to conditions such as obesity, diabetes, and metabolic syndrome. While these lesions are generally harmless, they can lead to discomfort or cosmetic concerns, prompting individuals to seek treatment. Traditional methods, including excision and cryotherapy, are frequently employed but come with certain risks. This study explores the use of Pachaieruvai, a traditional Siddha medicine, as an alternative treatment option. The subject of this report was a 50-year-old male who had a large skin tag on his gluteal region that had been growing for 15 years. Opting for Pachaieruvai over surgery, he applied the topical preparation for 10 days. Remarkably, the lesion was completely resolved within 15 days, and follow-up evaluations at two weeks, three weeks, and six months showed no recurrence or complications. Pachaieruvai comprises five ingredients recognized for their cytotoxic properties, including arsenic trioxide and copper sulfate, which may assist in targeting abnormal cell growth. However, the study's small sample size, absence of controlled trials, and potential safety concerns regarding arsenic necessitate further research to validate its efficacy and establish standardized treatment protocols.

## Introduction

Giant acrochordon, commonly known as a skin tag or fibroepithelial polyp, is a benign cutaneous neoplasm that appears as a large, soft, and pedunculated lesion. It is often found in areas prone to friction, such as the neck, axilla, groin, and intertriginous regions. Acrochordons are associated with comorbid conditions like obesity, diabetes, high cholesterol, insulin resistance, acromegaly, and metabolic syndrome, suggesting a link to lifestyle and metabolic factors [1,2]. They are typically harmless but can cause discomfort or cosmetic concern, leading individuals to seek removal. In most cases, a physical examination is enough for a diagnosis. However, there are rare instances where a histopathological examination might be needed [3]. Human papillomavirus (HPV) has been found in about 80% of skin tags, mainly subtypes 6 and 11. This suggests that HPV might play a role in the development of these lesions, although the exact mechanism is still not fully understood [4]. Skin tags are quite common, affecting around 25-30% of the general population, particularly among middle-aged and older adults. There’s a strong link between acrochordons and obesity; in fact, 74% of those with skin tags are either overweight or obese, and 24% meet the criteria for metabolic syndrome [5]. When it comes to treating skin tags, common methods include surgical excision and cryotherapy. However, it’s important to keep in mind that these treatments can come with risks, such as scarring, infection, and the possibility of the tags returning [6]. This report explores the exciting potential of Siddha medicine, an indigenous healing tradition from India. It specifically highlights Pachaieruvai as a promising and affordable alternative for managing acrochordon.

## Case presentation

A 50-year-old man came to our outpatient clinic with a noticeable, sizable, soft growth on the inner upper quadrant of his right gluteal region. This lesion has been around for 15 years, gradually getting larger over time. The patient mentioned that he hasn’t experienced any pain, itching, or bleeding; however, he has had occasional friction from clothing due to the size of the growth. His medical history is unremarkable, with no significant issues such as hypertension, diabetes, or other systemic diseases.

Upon examination, we identified the lesion as a large pedunculated skin tag, also known as an acrochordon, measuring about 12 cm in diameter. It was soft, non-tender, and could move freely, being attached to the skin by a narrow stalk. The skin covering the lesion was intact, showing no signs of inflammation, ulceration, or infection. The surrounding skin looked normal, and there were no other similar lesions found elsewhere on his body.

Diagnosis

The lesion was clinically diagnosed as a skin tag, or acrochordon, due to its characteristics as a large, soft, pedunculated, skin-colored growth. Both general and systemic examinations yielded no remarkable findings, and no other abnormalities were noted during the evaluation.

Management

The patient decided to seek treatment primarily due to ongoing irritation caused by friction with clothing. Rather than opting for surgical excision, he chose to pursue a traditional Siddha medicine approach, using Pachaieruvai, a topical preparation. Preparation typically involves several steps, including the purification and grinding of materials. Local applications are often crafted by traditional Siddha practitioners, who rely on specific instruments, such as stone mortars and pestles. The discussion section provides a detailed analysis of the various components involved in these preparations. Before starting the treatment, he provided written consent.

**Figure 1:**
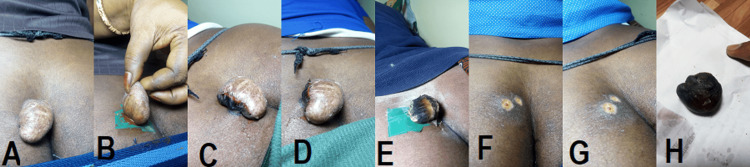
Progression of acrochordon stages. The images show: (A) before treatment, (B) day 1 of treatment, (C) day 3 of treatment, (D) day 5 of treatment, (E) day 8 of treatment, (F) day 12 of treatment, (G) day 15 of treatment, (H) separated skin tag progression of acrochordon.

The treatment plan involved applying Siddha medicine daily over the stalk of the skin tag for 10 consecutive days. During treatment, the patient experienced mild pain and a burning sensation at the application site, which lasted about 30 minutes after each application. These symptoms were temporary and resolved quickly. Interestingly, these reactions were viewed as positive signs indicating that the lesion was regressing. Ultimately, the treatment was successful, leading to the destruction of the growth within just 15 days. Figures 1A-1H show the progression of acrochordon.

Follow-up

The patient was reassessed two weeks after completing the treatment, and the site of the lesion had healed remarkably well, showing no signs of infection or any residual growth. During the three to six-month follow-up, there was no evidence of recurrence or any adverse effects, which confirmed the effectiveness and safety of the treatment regimen involving Pachaieruvai. The patient remained symptom-free, and no complications were reported, underscoring the success of this alternative approach in managing a large acrochordon.

## Discussion

This case highlights the successful application of Pachaieruvai, a traditional Siddha medicinal preparation, as an effective alternative therapy for treating a large acrochordon. Rooted in ancient Siddha texts, Pachaieruvai is made up of the following five key ingredients: vellaipadanam (arsenic trioxide), aridharam (arsenic trisulfide), thurusu (copper sulfate), karchunnam (calcium carbonate), and kungiliyam (resin from *Shorea robusta*) [7]. Each of these components brings unique pharmacological properties to the table. Research has shown that vellaipadanam, commonly known as arsenic trioxide and a vital part of Pachaieruvai, possesses significant anti-glioma and antiviral effects. Studies suggest that it can reduce the growth of glioma cells while promoting programmed cell death [8]. Both vellaipadanam and aridharam have demonstrated powerful cytotoxic effects, aiding in the induction of apoptosis and the inhibition of tumor growth [9]. Thurusu, or copper sulfate, is notable for its cytotoxic properties that also inhibit tumor growth and exhibit antiviral effects. Its mechanism involves the generation of reactive oxygen species (ROS), which trigger cytotoxic reactions that compromise cellular integrity and prevent the growth of abnormal cells [10-12]. Karchunnam plays a role in further suppressing abnormal cell growth, while kungiliyam, a resin extracted from *Shorea robusta*, shows strong cytotoxic properties against abnormal cellular growth. Research indicates that extracts from *Shorea robusta *can hinder the proliferation of cancer cells. Notably, robustic acid derivatives found in this resin have exhibited potent cytotoxic effects against various cancer cell lines, including HL-60 (human leukemia) and HepG2 (human hepatoblastoma) [13-15].

When combined, these five ingredients work synergistically to specifically target the abnormal cells associated with acrochordons. The cytotoxic effects of vellaipadanam, aridharam, thurusu, and kungiliyam collaborate to promote cell death and prevent the excessive growth of these abnormal cells. Calcium carbonate plays a vital role by creating an optimal environment that enhances the effectiveness of these active ingredients. Together, this unique blend not only helps to reduce the size of skin tags but can also lead to their complete elimination by directly attacking the cells responsible for their growth, resulting in noticeable shrinkage or even disappearance of the lesion.

Limitations of the study

This report has several limitations that impact the generalizability and reliability of the findings regarding Pachaieruvai for treating acrochordons. The lack of a larger sample size restricts our ability to draw robust conclusions about its effectiveness and safety across a broader population. Additionally, the absence of controlled trials makes it challenging to determine whether the observed results are genuinely due to the Siddha medicine or influenced by natural regression or other factors. Although the patient showed no recurrence at three and six months, a longer follow-up period is essential to confirm the treatment's long-term efficacy and safety. The subjective reports of mild discomfort also highlight variability in patient experiences, as there was no standardized measurement for these outcomes. Furthermore, potential biases in reporting outcomes and the lack of objective data, such as histological analyses, weaken the findings. The exact mechanisms by which Pachaieruvai's ingredients act on acrochordons remain unclear, necessitating further biochemical research. The study also emphasizes the need for standardization in Siddha medicine, as preparation and dosage can vary significantly among practitioners. Lastly, while no immediate adverse effects were noted, the inclusion of arsenic-based compounds raises safety concerns that warrant further investigation, particularly regarding the long-term risks associated with arsenic exposure.

## Conclusions

This study demonstrates the successful treatment of a large acrochordon using Pachaieruvai, a traditional Siddha medicine. Complete resolution occurred within two weeks with no recurrence at six-month follow-up. The cost-effective treatment produced excellent cosmetic results with minimal side effects. While promising, this single case necessitates further research including controlled clinical trials with larger cohorts, standardized preparation protocols, comparative studies with conventional treatments, and safety assessments of arsenic compounds. Scientific validation of traditional medicines like Pachaieruvai is essential for their integration into modern dermatological practice and recognition of Siddha medicine's potential value in treating skin conditions.
